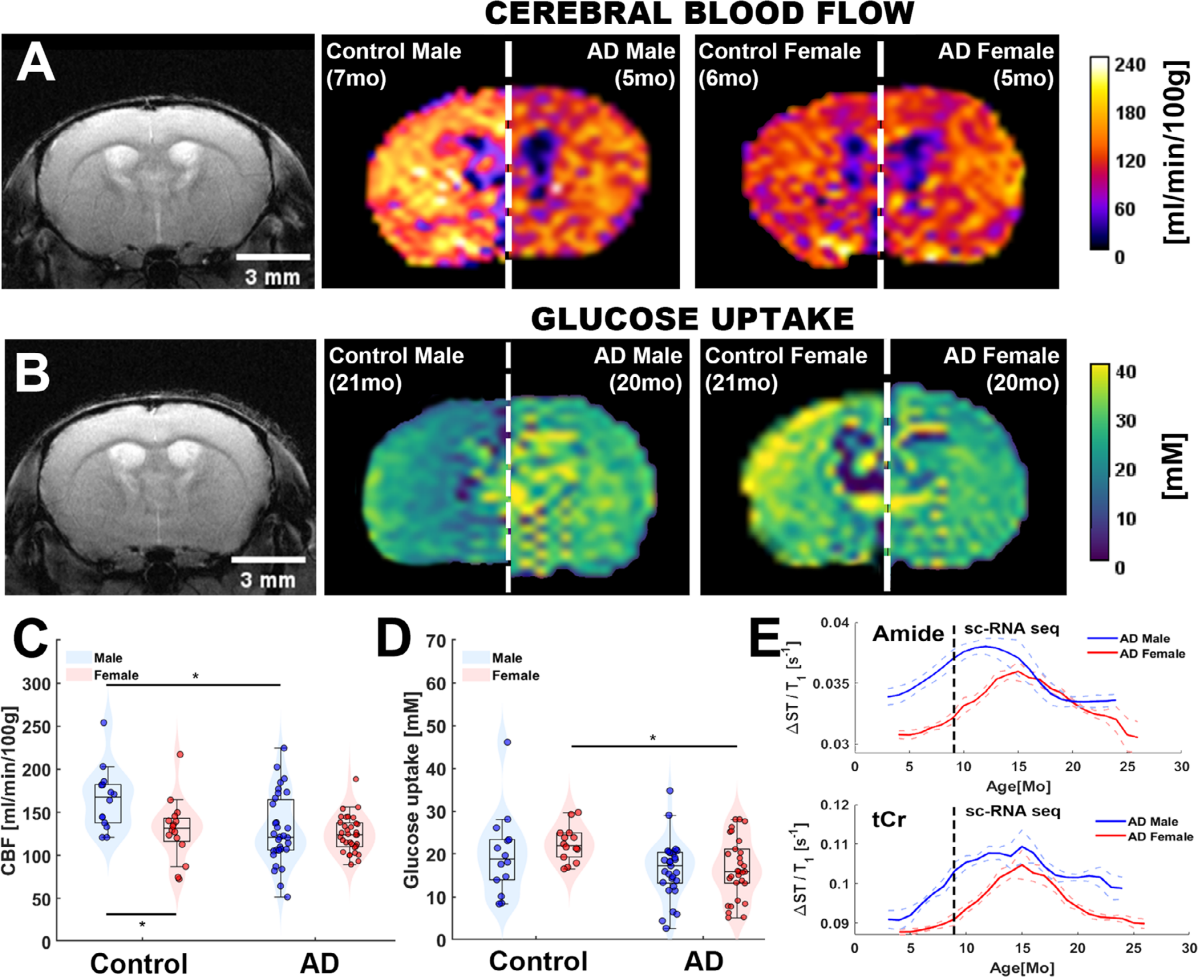# Sex‐specific metabolic and vascular brain aging in preclinical models of dementia

**DOI:** 10.1002/alz70856_102243

**Published:** 2025-12-25

**Authors:** Yifan Zhao, Tao Jin, Nicholas F Fitz, Yi Lu, Radosveta Koldamova, Bistra Iordanova

**Affiliations:** ^1^ University of Pittsburgh, Pittsburgh, PA, USA; ^2^ University of pittsburgh, PITTSBURGH, PA, USA

## Abstract

**Background:**

Alzheimer's disease (AD) begins with subtle changes in asymptomatic people and eventually leads to clinical symptoms and dementia. On cellular level, in addition to neurodegeneration, microglia and astrocytes are central to AD etiology. Vascular contributions such as cerebral amyloid angiopathy (CAA), microbleeds, hypoperfusion and hypometabolism are common for both women and men with AD, however the pathways, severity and presentation appear to be sex‐specific. In this study, we combined vascular and metabolic brain imaging with single‐cell transcriptomics to relate functional aspects of neurovascular and metabolic resilience to cell‐specific molecular pathways.

**Method:**

We studied mice across the lifespan, 4–24 months. We used APP/PS1 (*n* = 64, 33 females and 31 males) and B6C3 controls (*n* = 36, 17 females and 19 males). We measured blood flow, brain metabolites and glucose uptake using quantitative cerebral blood flow (qCBF), and chemical exchange saturation transfer (CEST) MRI at 9.4Tesla. We quantified the amyloid plaques and CAA load in histological sections. Single‐cell mRNAseq focused on microglia and astrocytes.

**Result:**

Control mice had higher CBF (*p* = 0.0283), glucose uptake (*p* = 0.009) and magnetization transfer (MT, *p* = 0.005) than AD. The CBF changes appeared earlier than the glucose changes and each were driven by different sex (Figure 1A,B). We identified sex‐differences in CBF, glucose uptake and brain metabolites. CBF was higher in male controls (*p* = 0.0099) than females and this sex difference disappeared in AD (Figure 1C). The lower CBF of AD males compared to control males had biggest drop at 10 months (*p* = 0.0066). Surprisingly, this CBF difference was not present between the control and AD females. In contrast, glucose metabolism was lower in AD females comparing to control females (*p* = 0.0012) but we saw no difference between males (Figure 1D). AD female mice had lower amide (*p* = 0.0211) and creatine (*p* = 0.0167) than AD males with largest change after 10 months (Figure 1E), and no sex differences in controls. We saw gene expression changes in pathways of cellular respiration, angiogenesis, inflammation, lipid metabolism, and creatine transport.

**Conclusion:**

We present integrative perspective on vascular and neuroenergetic function in preclinical dementia models. Our approach combines multiscale imaging and transcriptomics to construct trajectories of sex‐specific healthy and pathological brain aging.